# Supplementary material for: Anisotropic electrical resistance in mesoscopic LaAlO3/SrTiO3 devices with individual domain walls
Source: Sci Rep. 2017 Mar 15;7:44361. doi: 10.1038/srep44361 (PMC5353628; doi:10.1038/srep44361)
Supplement: Supplemental Information [file srep44361-s1.pdf]

Anisotropic electrical resistance in mesoscopic $LaAlO_3/SrTiO_3$ devices with domain walls

Nicholas J. Goble[1], Richard Akrobetu[2], Hicham Zaid[3], Sukrit Sucharitakul[1], Marie-Hélène Berger[3], Alp Sehirlioglu[2], and Xuan P. A. Gao[1,*]

[1]Department of Physics, Case Western Reserve University, Cleveland, Ohio 44106, USA
[2]Department of Materials Science and Engineering, Case Western Reserve University, Cleveland, Ohio 44106, USA
[3]MINES Paris Tech, PSL Research University, MAT - Centre des matériaux, CNRS UMR 7633, BP 87 91003 Evry, France

Supplementary Information

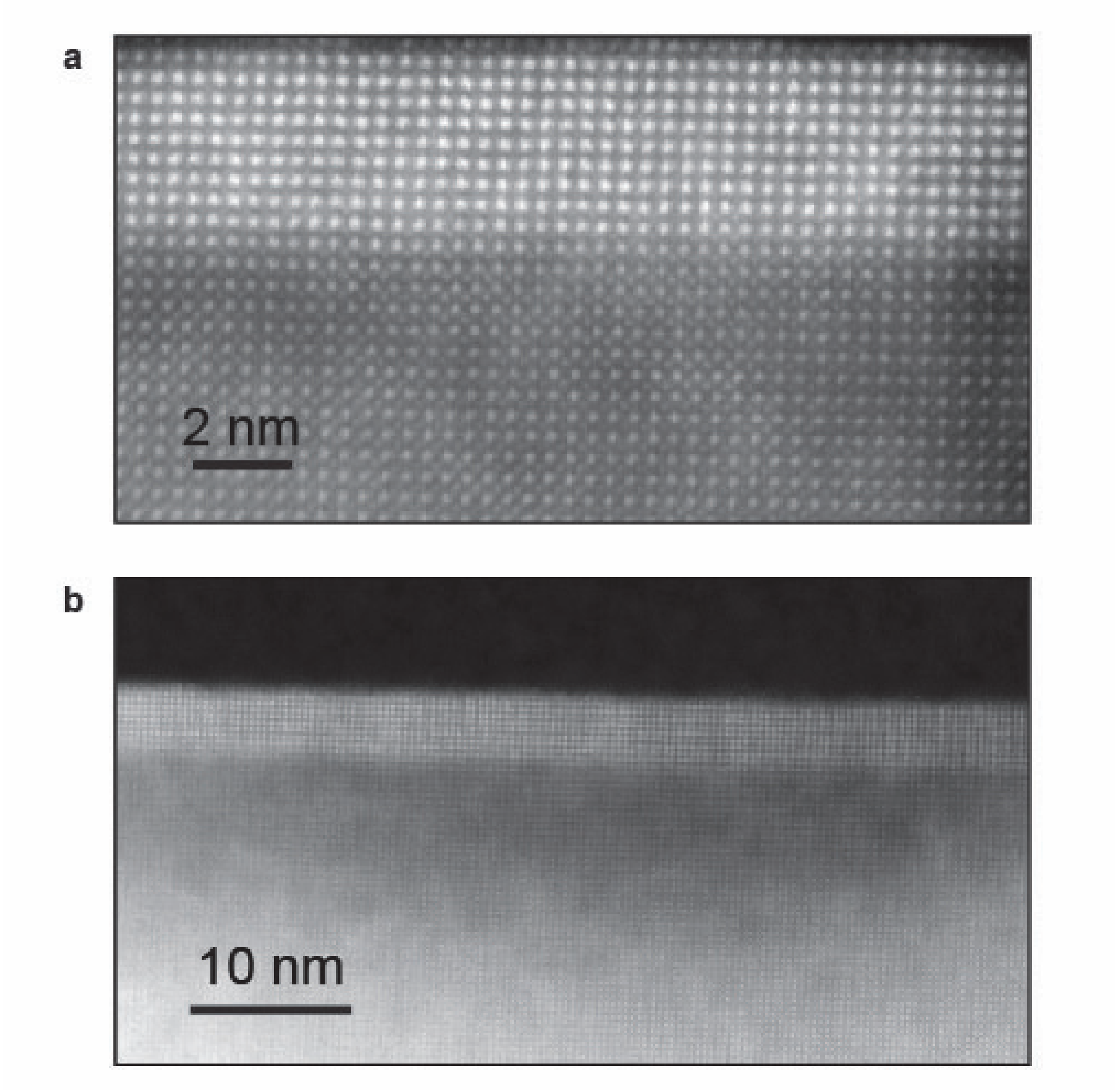

Figure S1. LAO/STO STEM images. Scanning transmission electron microscopy (STEM) images taken on a 10 unit cell thick LAO/STO sample grown at an oxygen partial pressure of 10^{-4} Torr. Brighter spots on the top of the figures are La atoms, while

dimmer spots below the interface indicate Sr atoms. A clear interface forms between LAO and TiO_2-terminated STO.

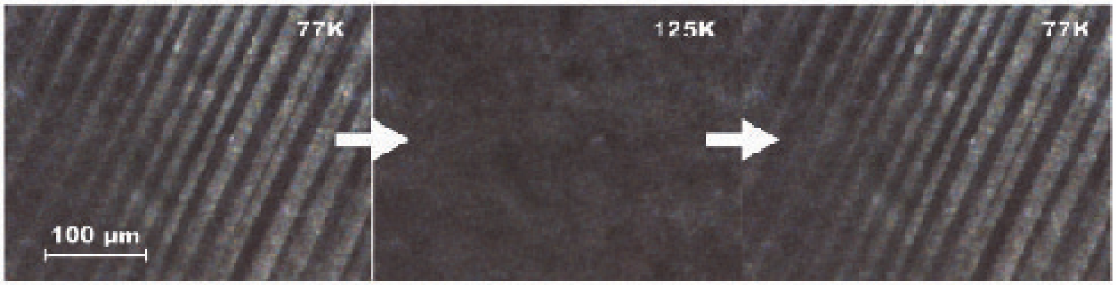

Figure S2. Striped tetragonal domain imaging through thermal cycling. Multiple LAO/STO samples were imaged through the structural phase transition prior to patterning and etching. Samples were heated from 77 K to 125 K and subsequently cooled back down to 77 K. We observe no change in the domain wall configuration due to thermal cycling through the cubic-to-tetragonal phase transition.

Sample number	LAO thickness	O_2 partial pressure	Domain wall∠	$R_{parallel}$ / R_{perp} (10K or 20K)	Area	Estimated carrier density (10 K)
10.1	10 u.c.	10^{-4}Torr	90°	Up to 70,000	40x40 μm^2	3.64×10^{13} cm^{-2}
10.2	10 u.c.	10^{-4}Torr	90°	16	40x40 μm^2	2.59×10^{13} cm^{-2}
10.3	10 u.c.	10^{-4}Torr	80°	10	20x20 μm^2	3.21×10^{13} cm^{-2}
10.4	10 u.c.	10^{-4}Torr	45°	1.45	20x20 μm^2	3.49×10^{13} cm^{-2}
10.5	10 u.c.	10^{-5}Torr	none	1.25	10x10 μm^2	2.50×10^{15} cm^{-2}
10.6	10 u.c.	10^{-4}Torr	none	1.35	20x20 μm^2	n/a
8.1	8 u.c.	10^{-5}Torr	90°	15	30x30 μm^2	n/a

Table S1. Collection of patterned and measured samples. All seven samples studied were grown using pulsed laser deposition. A LAO thickness of 10 u.c. provided reliable interfacial conduction in all growth conditions, though a sample with 8 u.c. LAO thickness was studied as well, providing comparable results. Samples grown at a lower O_2 partial pressure showed substantially higher carrier densities due to the increase in oxygen vacancies in the STO crystal. Sample 10.1 was patterned with eight contacts, allowing for multiple measurement configurations across the same domain wall with different anisotropy ratios that could reach as high as 70000.

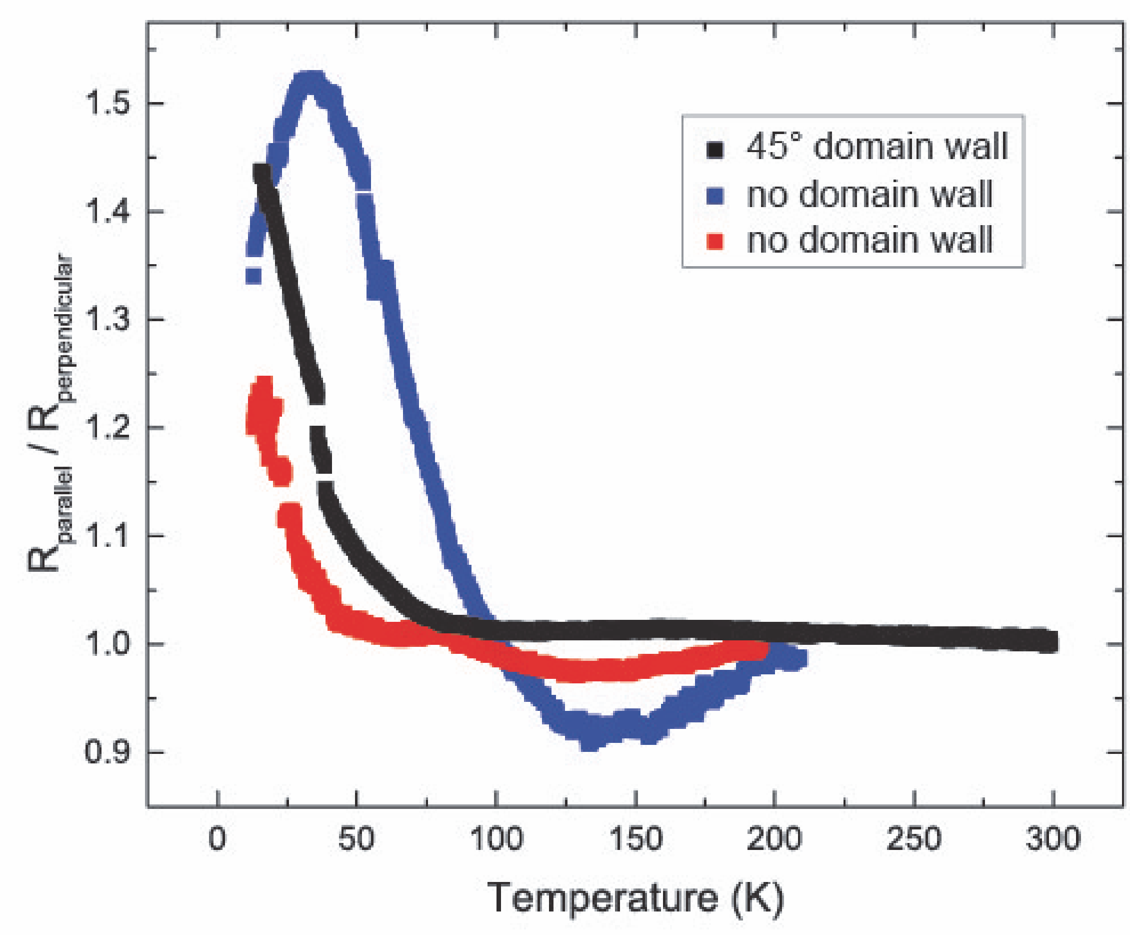

Figure S3. Temperature dependent anisotropy for LAO/STO samples with no domain wall or a 45° domain wall. The strength of anisotropy is minimal in samples with no domain walls. The relative difference in resistance between orthogonal measurement directions is less than 1.5 at low temperatures.

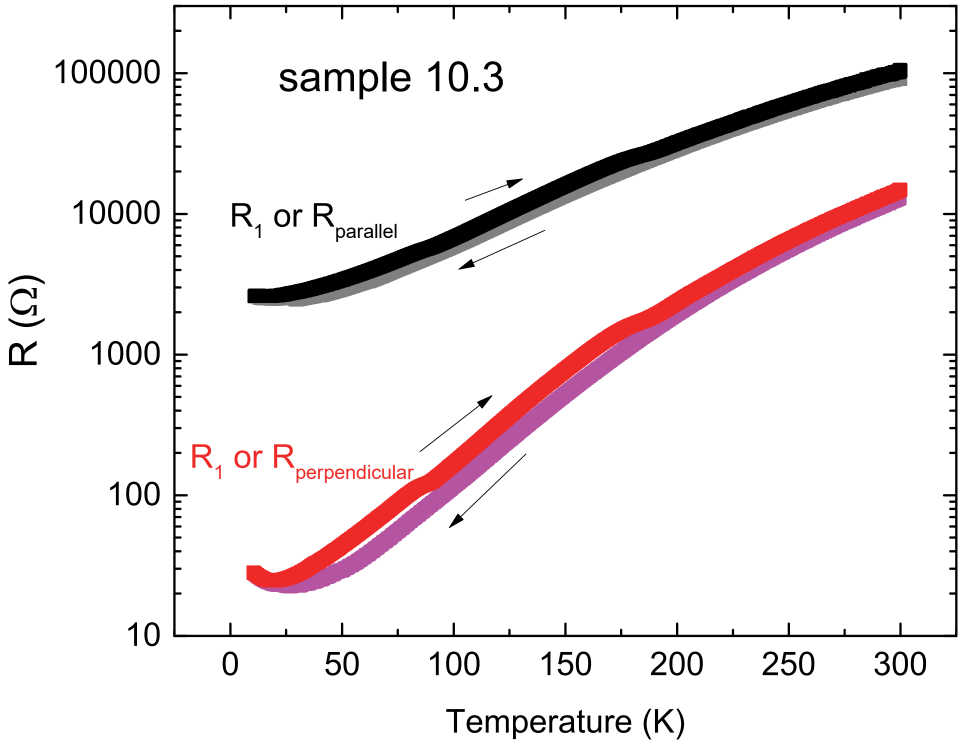

Figure S4. Temperature dependent resistance for LAO/STO sample 10.3 measured along two directions, showing the increasing anisotropy at low temperatures and a pre-existing anisotropy at 300K.

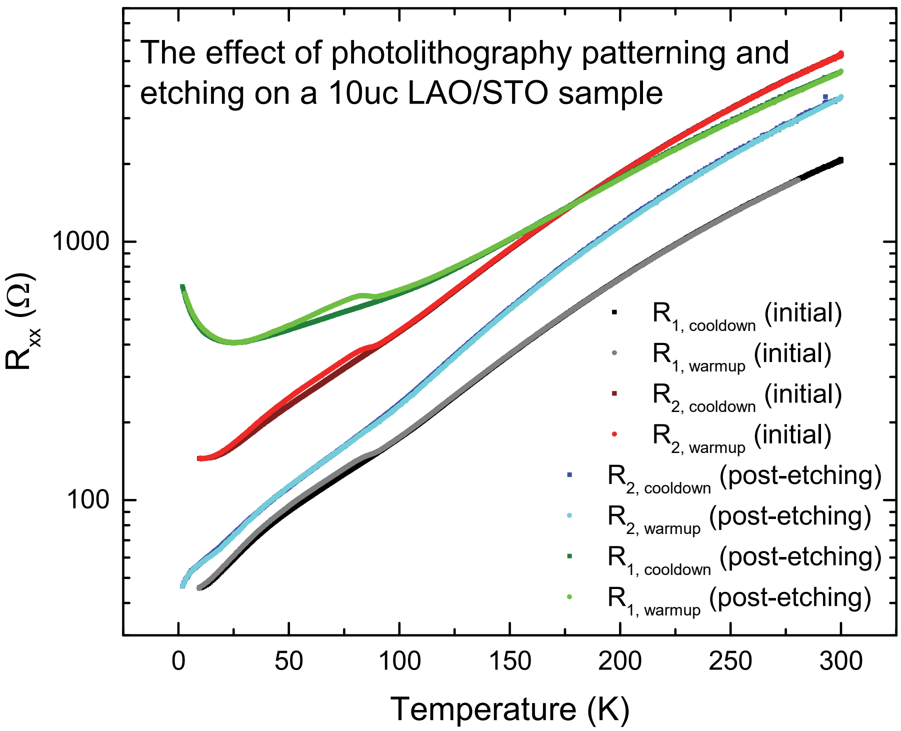

Figure S5. Temperature dependent resistance R_1 and R_2 of a 10uc LAO/STO sample measured along two directions at 90 degrees from each other, before (black and red dots) and after (green and blue) the photolithography patterning and wet etching. It is seen that the sample resistances before/after patterning and etching are similar at room T. At the same time, after the patterning, the difference between R_1 and R_2 at room temperature became smaller, indicating a more homogenous device after etching.

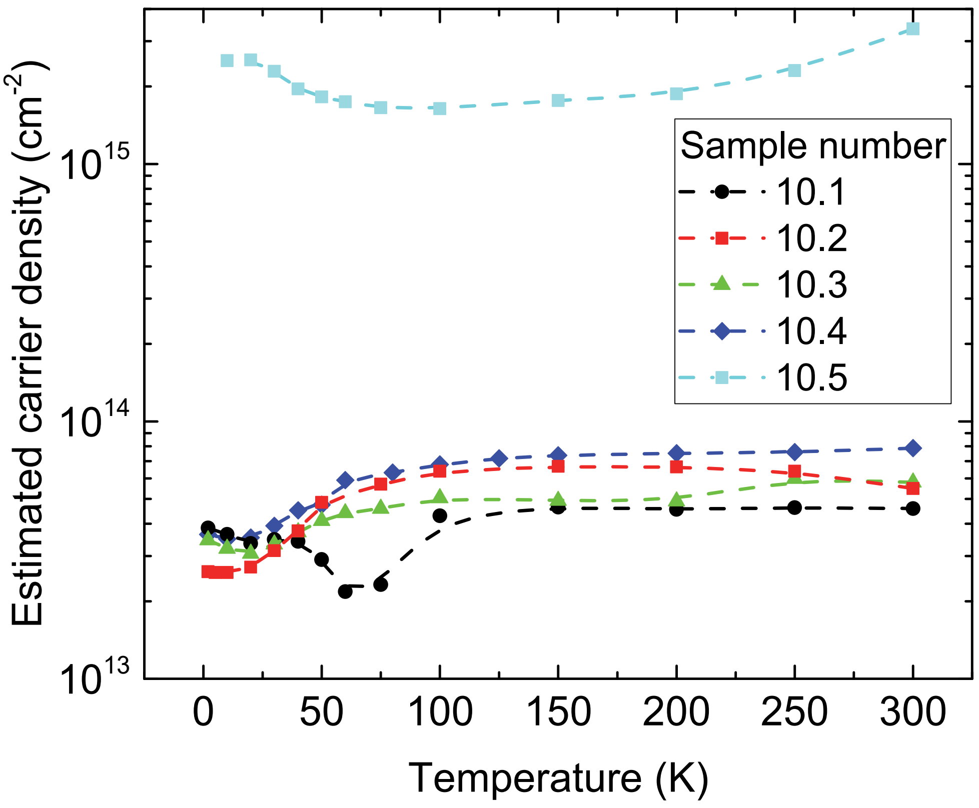

Figure S6. Temperature dependent carrier density for LAO/STO samples. Hall carrier density is shown throughout a temperature range of 300 K to 2 K. Values were estimated from Hall coefficients, which were collected in a ±2 T magnetic field. LAO/STO samples (#10.1-10.4) grown at a higher O_2 partial pressure (10^{-4} Torr) have substantially less charge carriers than sample 10.5 which was grown at a lower O_2 partial pressure (10^{-5} Torr).
